# Weaknesses and strengths in the emergency response and management of the first mpox case in The Gambia

**DOI:** 10.4102/jphia.v17i1.1648

**Published:** 2026-03-09

**Authors:** Sheikh O. Bittaye, Lamin Manneh, Morikebba Danso, Sheriffo Jagne, Amadou W. Jallow, Ya Fatou B.M. Jobe, Mary Bobb, Ebrima K. Jallow, Momodou Kalisa, Kebba Jobarteh, Modou L. Sanneh, Haddijatou Allen, Ifeanyi L. Udenweze, Pius Ononigwe, Abdoulie Badjan, Mustapha Bittaye, Momodou T. Nyassi

**Affiliations:** 1 Department of Internal Medicine, Faculty of Medicine, University of The Gambia, Banjul, The Gambia; 2 Ministry of Health, Banjul, The Gambia; 3 Department of Internal Medicine, Faculty of Medicine, Edward Francis Small Teaching Hospital, Banjul, The Gambia; 4 National Public Health Laboratories, Kotu, The Gambia; 5 Department of Epidemiology and Disease Control, Ministry of Health, Banjul, The Gambia; 6 World Health Organisation, Kotu, The Gambia; 7 Africa Centre for Disease Control and Prevention, Banjul, The Gambia; 8 Department of Pathology, Faculty of Medicine, Edward Francis Small Teaching Hospital, Banjul, The Gambia; 9 Department of Pathology, Faculty of Medicine, University of The Gambia, Banjul, The Gambia; 10 Department of Obstetrics and Gynaecology, Faculty of Medicine, Edward Francis Small Teaching Hospital, Banjul, The Gambia; 11 Department of Obstetrics and Gynaecology, Faculty of Medicine, University of The Gambia, Banjul, The Gambia

**Keywords:** mpox, Gambia, case study, emergency response, strengths, weaknesses

## Abstract

Mpox is a zoonotic virus that can infect humans and animals. The director general of the World Health Organization (WHO) declared the mpox outbreak a public health emergency of international concern on 14 August 2024, with the greatest burden in Africa. The Gambia registered its first case of mpox on 18 July 2025. This case study, therefore, assesses the weaknesses and strengths in the emergency response and management of the first mpox case in The Gambia. The patient is a 26-year-old female Gambian, who presented with a two-day history of a skin rash which was associated with fever, headache and myalgia. This patient was seen at the health centre, and swab samples were collected for mpox testing before she returned home. The samples were delivered to the National Public Health Laboratories (NPHL) a day later, and the polymerase chain reaction tests were conducted 8 days later, which confirmed the presence of mpox virus infection. The confirmed mpox case initially presented challenges with compliance, as the patient could not be readily located for isolation and treatment. However, through the coordinated efforts of the police, mobile operators, the village health worker (VHW), field investigators, surveillance officers, public health officers, regional health directorate staff, the head of the village or community, and the nurse at the Fajikunda Health Centre (FJKHC), the case was successfully traced. The assessment of the emergency response and management of the first mpox case in The Gambia revealed notable strengths and weaknesses. Surveillance efforts at the primary healthcare level were effective, leading to the detection of the case and a well-coordinated overall response. However, significant challenges emerged in the laboratory analysis of collected samples, including delays in processing as a result of an unreliable electricity supply and gaps in appropriate infection prevention and control measures.

## Introduction

Mpox is a zoonotic virus that can infect humans and animals. It was initially discovered in a group of cynomolgus macaques (*Macaca fascicularis*) kept in captivity in 1958.^1^ It is a double-stranded deoxyribonucleic acid (DNA) virus that is part of the *Orthopoxvirus* genus within the Poxviridae family.^2,3^

The director general of the World Health Organization (WHO) declared the mpox outbreak as a public health emergency of international concern on 14 August 2024.^4^ By epidemiological week 39 of 2024, 35 525 cases had been reported across 16 African Union Member States, with 6754 mpox cases confirmed and 996 deaths.^5^ Mpox is endemic in several African countries, especially the western and central African countries, and currently, the infection has also spread in non-endemic countries.^6,7^

The global response to this mpox outbreak involved coordinated efforts from various stakeholders and individual governments. Nonetheless, numerous African nations face persistent public health difficulties in addressing infectious diseases that are of significant public health concern, particularly in regions with inadequate healthcare or public health systems.^8^ The Gambia is a resource-limited country, and it registered its first mpox case on 18 July 2025. Since the confirmation of the first case, a total of 99 suspected cases were recorded, but all were negative for mpox. This case study, therefore, assesses the weaknesses and strengths of the emergency response and management of the first mpox case in The Gambia.

## Timeline of emergency response activities for the first mpox case in The Gambia

A 26-year-old female Gambian residing in Western Region 1 (WR1) presented with a 2-day history of a skin rash, which was associated with fever, headache and myalgia. The skin rash was vesiculopapular and started on the back and then spread to the face, trunk and other parts of the body, including the mouth and genitalia. As the severity of the symptoms progressed, she visited a nearby pharmacy and was referred to the Fajikunda Health Centre. She was examined and given medication (oral cloxacillin 500 mg 6 hourly for 5 days, oral Piriton 4 mg 8 hourly for 5 days, oral paracetamol 1 g 8 hourly for 3 days, sulphur soap bath 12 hourly and permethrin ointment to apply 12 hourly). The lesion material (swabs) was collected for mpox testing, and the patient was allowed to go home. The samples were delivered to the National Public Health Laboratories (NPHL) a day later, and the polymerase chain reaction (PCR) tests were conducted 8 days later, which confirmed mpox virus infection.

The Epidemiology and Disease Control (EDC) unit was informed about the confirmed mpox case a day after, and the rapid response team of the Western Region I was deployed at the same time to commence outbreak response activities, including case investigation, contact identification, listing and tracing, and community engagement. The team comprised field investigators, surveillance officers, public health officers, village health workers (VHWs) and Regional Health Directorate staff. The investigation team, led by the VHW, converged at the head of the village and/or community compound at around 11:45. At approximately 24:05, the team proceeded to the community nursery school, where the case gave us her address. After initial contact was attempted through repeated calls, the case refused to engage or cooperate with the team. Following this, the team, along with VHWs, proceeded to seek options for reaching her through the police post. The team was then referred to the main police station for further action. The team arrived there at approximately 13:20. The surveillance officer from the Regional Health Directorate introduced the team and briefed the officers on the public health threat posed by the case and the need for support. A file was then opened at the Criminal Investigation Department (CID) by the police, and they recommended applying for a call printout of the case’s mobile number to help trace her current location. At around 13:34, one of the officers placed a call to the case, during which she categorically stated that she was not interested in meeting any health or police official and claimed that she had already travelled out of the village and was on traditional treatment. She further emphasised that her health is her personal responsibility and refused further dialogue. Given her refusal, the police advised the health team to escalate the matter to higher health authorities for coordination with security services to facilitate access to telecommunications data. Following this, the investigation team submitted a request to obtain a printout of the patient’s phone record, which would aid in tracing her location and ensuring the appropriate public health intervention. Two days later, her phone was tracked to be 35 km from where the patient was residing, but the patient was not found there. The team then further engaged the nurse who first saw the patient. Because of the trust and confidence in the nurse, the patient was finally convinced. She was then invited to a meeting with the director of health services, the programme manager of the EDC Unit, the nurse who saw her at the health centre and other senior health staff at the EDC office. She was then persuaded and was sent to the national treatment centre for treatment the following day for isolation and treatment.

The field investigators, comprising the VHWs and public health officers, listed 18 contacts. The contacts were followed up by daily phone calls and three times weekly visits by VHWs and public health officers. They were monitored for 21 days, but none developed mpox symptoms.

**FIGURE 1:**
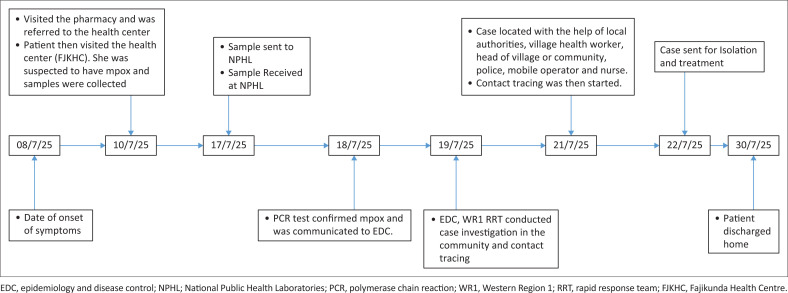
Timeline of emergency response activities from the onset of symptoms to the discharge of the patient from the national treatment centre.

At the national treatment centre, she was managed by a multidisciplinary team which included doctors, nurses, infection prevention and control (IPC) personnel, and psychosocial and/or mental health team. She was put on ampicillin–cloxacillin 500 mg 6 hourly, paracetamol 1 g 8 hourly and triple action cream. She was also given psychosocial support daily. Twenty-one days after the onset of symptoms, the patient was clinically stable, all lesions had resolved, and she was afebrile. She was subsequently discharged and allowed to return home with an appointment scheduled for follow-up (Figure 1).

## Discussion

This case study highlights the weaknesses and strengths during the early phase of the mpox outbreak response in The Gambia. These findings suggest a good surveillance system at the primary healthcare level, well-coordinated response from the national and regional levels to the community level in the tracing and investigation of the case, effective contact tracing, and efficient multidisciplinary approach in the management of the patient. There was, however, a delay in turnaround sample processing time as a result of unreliable electricity supply and the lack of appropriate IPC measures in place to prevent transmission of infection at the health facility and community levels.

The application of proper and efficient IPC measures is critical to breaking the chain of mpox transmission. Central to these measures is the avoidance of direct person-to-person contact with suspected or confirmed cases, as the virus spread primarily through through close physical contact with lesion materials or contaminated objects. Within healthcare settings, strict compliance with standard precautions, including the use of personal protective equipment (PPE), safe handling of clinical specimens, proper waste management and environmental disinfection, is vital to protecting health workers and other patients. In community settings, raising awareness about safe practices, such as limiting physical contact, isolating symptomatic individuals and promoting hand hygiene, is equally important to prevent household and community spread. Ensuring that both healthcare providers and community members consistently apply these measures significantly reduces the risk of outbreaks and contributes to the effective control of mpox. The United States CDC and WHO recommend isolation of suspected or confirmed mpox cases and social distancing.^9^ In this case, there was a high index of suspicion for mpox, but unfortunately, the patient was not isolated but left to go home. This move increases the risk of the spread of mpox infection in both the health facility and the community. However, once the patient was confirmed positive, an isolation area was identified at the health facility, and staff also had refresher training on IPC. At the community level, the family was educated about mpox, advised on IPC measures such as hand washing, separation, drying and ironing of beddings and clothes of the index case, and regular follow-up of contacts.

The confirmation of mpox relies on real-time PCR, a laborious method that requires a highly sophisticated thermal cycler, which makes it inappropriate or difficult for widespread use in resource-limited countries.^10^ In this case, samples were collected and delivered the next day to the NPHL, but it took the laboratory 8 days to conduct PCR analyses as a result of unreliable electricity supply. This incident indicates that, despite the availability of real-time PCR, several laboratory-related challenges, such as difficulties in sample transportation and delays in processing caused by limited or unreliable electricity supply, can contribute to delays in the detection of mpox in resource-constrained settings.

Integrated disease surveillance and response (IDSR) aims to establish a national disease surveillance system with capacities to prepare, detect and effectively respond to high-priority communicable and non-communicable diseases and other events of public health importance.^11,12^ The Gambia has a very strong integrated surveillance system and has trained more than 500 healthcare workers across the country on the Third Edition of the IDSR Technical Guidelines. In this case, as a result of the high index of suspicion of mpox, the patient was referred from a neighbourhood pharmacy to the nearest health facility, where samples were collected for mpox testing. The contacts of the index case were monitored with daily phone calls and three times weekly visits by VHWs and public health officers throughout the disease’s incubation period, and none developed symptoms by the end of the follow-up.

In 2019, The Gambia established a Public Health Emergency Operation Centre (PHEOC), which serves as a hub that helps in the effective coordination of information and resources during the management of public health emergencies.^13^ Even though The Gambia is yet to have a legally existing PHEOC, there are existing structures from the national to the regional levels. Once the case was confirmed positive, the national PHEOC was activated at Level 2 to ensure a coordinated and effective response, enabling the timely mobilisation of resources and implementation of measures to protect the health and safety of the population. The incident management team under the Directorate of Health Services, Ministry of Health, in collaboration with the regional rapid response team, the neighbourhood pharmacies, the first clinician who had contact with the patient at the health facility, the police, mobile operators, village development committee members, head of the village or community, and VHWs also went into action to investigate the case in the community. The case was traced, an appropriate case investigation form filled and then admitted to the treatment centre. This case confirms the importance of collaboration with the primary healthcare system, local government officials, security officers, other health stakeholders and mobile operators in response to public health emergencies. Response interventions such as risk communication and community engagement using mass media, social media, Civil Society Organisations (CSOs) and/or Non-Governmental Organisations (NGOs), and mobile operators were also intensified. World Health Organization, Africa CDC, United Nations Children Fund (UNICEF), The Gambia Red Cross country offices, CSOs, department of livestock, and security officers among others worked closely with the Incident Management Team and the Ministry of Health in supporting response interventions particularly in coordination and also response pillar activities in the form of finance and/or technical support, such as enhanced surveillance at community levels through active case search and contact tracing, training of healthcare workers as well as community volunteers (signal detectors) on mpox surveillance, risk communication and community engagement, and isolation of any suspected case for treatment. This activity also reflects the response approach in Africa Continental mpox Incident Management Support Team (IMST), where partners, including WHO, Africa CDC and UNICEF, provide coordination and support to member states through the African Union (AU), thereby ensuring strategic, cost-effective and impactful response.^5^ No travel advice was released or entry restrictions, but the eight points of entry into The Gambia were put on high alert to actively search for suspected cases among travellers entering the country.

The treatment of mpox primarily focuses on managing symptoms and preventing complications.^14,15^ The management of the case involved a multidisciplinary team, which included medical doctors, nurses, IPC personnel, and a psychosocial and/or mental health nurse. The medical doctors reviewed the patient on a daily basis and prescribed medications when necessary. The nurses were responsible for vital sign checking and administration of prescribed medications. The psychosocial and/or mental health nurse offered daily sessions of psychotherapy, and the IPC personnel ensured that proper IPC procedures were followed and implemented within the facility. This patient was put on antibiotics and analgesics. The patient’s condition improved, and she was discharged. This case emphasises the need for a multidisciplinary approach in the management of mpox patients.

The response to this first mpox case in The Gambia is unique in the sense that it presented enormous challenges in compliance for tracing, case investigation and isolation when compared to that for other first mpox cases in other countries.^16,17,18^ However, with a well-coordinated search exercise at the community level through the use of community structures (VHWs and head of the village and/or community), police, mobile operators and healthcare workers, the case was successfully traced. There was also a delay in sample turnaround time as a result of unreliable electricity and inadequate IPC measures in the health facility and community. In contrast, countries like Pakistan had functional PCR machines but lacked reagents at the time of detecting their first case.^16^ Oman and Trinidad and Tobago seem to have had adequate diagnostic capabilities at the time of diagnosing their first case.^17,18^ This situation suggests that each country’s emergency response to its first cases may differ, and thus identifying their weaknesses and strengths of the emergency response to their first cases will help to improve their countries’ responses in future cases.

## Conclusion

These findings suggest an effective primary healthcare surveillance system, a coordinated response at the national, regional and local level and an effective multidisciplinary approach in the management of this patient. Nonetheless, there was a delay in sample turnaround time as a result of unreliable electricity supply and inefficient IPC measures to prevent transmission at both the health facility and the community levels.
